# Debating personal health budgets

**DOI:** 10.1192/pb.bp.114.048827

**Published:** 2016-02

**Authors:** Vidhya Alakeson, Jed Boardman, Billy Boland, Helen Crimlisk, Charlotte Harrison, Steve Iliffe, Masood Khan, Rory O'Shea, Janet Patterson

**Affiliations:** 1 NHS England; 2 Institute of Psychiatry, King's College London; 3 Hertfordshire Partnership University NHS Foundation Trust; 4 Sheffield Health and Social Care Foundation Trust; 5 South West London and St George's Mental Health NHS Trust; 6 University College London; 7 Royal College of Psychiatrists; 8 Northumberland, Tyne and Wear NHS Trust; 9 Oxford Health NHS Foundation Trust

## Abstract

Personal health budgets (PHBs) were piloted in the National Health Service (NHS) in England between 2009 and 2012 and were found to have greater positive effects on quality of life and psychological well-being for those with mental health problems than commissioned service, as well as reducing their use of unplanned care. The government intends to extend PHBs in England for long-term conditions, including mental health, from April 2015. Given the importance of engaging clinicians in the next phase of PHB development, we provide an overview of the approach, synthesise the evidence from the national pilot and debate some of the opportunities and challenges. Balancing individual choice and recovery with concerns for risk, equity and the sustainability of existing community services is the central tension underpinning this innovation in mental health service delivery.

Direct payments and personal budgets in social care have been introduced as part of a move towards the personalisation of care,whereby a person is able to choose the services best placed to meet their unique needs. Their equivalent in healthcare, personal health budgets (PHBs; www.personalhealthbudgets.england.nhs.uk), are not yet widely available, but has been subject to pilot testing in England and, since October 2014, all individuals who are eligible for continuing healthcare have the right to have a PHB.^1^ Around 56 000 people are eligible for continuing healthcare. These people have the highest level of ongoing health and support needs and the National Health Service (NHS) pays for their health and social care. Adults with mental health conditions are not often eligible for continuing health care because they often do not have high nursing care-type needs, but older adults with dementia are more likely to qualify as are some people with intellectual disabilities. The roll-out of PHBs for other groups, such as those with mental health problems, is currently at the discretion of local decision makers, although NHS England expects clinical commissioning groups to lead a major expansion of PHBs in 2015-2016. The policy and plans for PHBs are limited to the NHS in England.

Many clinicians may be unaware of the developments in PHBs^2^ and only a few psychiatrists were involved in the national pilot which suggested that PHBs may be of value to people with mental health conditions. The purpose of this paper is to introduce the concept of PHBs and to highlight some of the opportunities and challenges that the use of PHBs presents. Our intention is to extend the debate on the use of PHBs and their value in mental health services.

## What is a personal health budget?

A personal health budget is an individual allocation of NHS resources that can be used to meet identified health and well-being needs in possibly new and innovative ways outside of traditionally commissioned services. The national evaluation identifies a wide range of purchases made with a PHB, from traditional clinical services such as therapies and nursing care, to social care-related services such as meal preparation and social activities, to well-being services such as gym memberships, computers and art classes.

A PHB is not intended to cover all aspects of NHS care: in-patient care, emergency services, general practitioner (GP) services and pharmaceuticals are all excluded, and certain goods and services are prohibited. PHBs are focused on meeting ongoing needs where bringing together the lived experience of individuals and the learned expertise of clinical professionals can improve the quality and outcomes of care. In this respect, they have much in common with other approaches to personalising the management of long-term conditions such as the house of care model^3^ and shared decision-making.

At the centre of a PHB is a care plan which is developed by the individual in conjunction with their clinical team and signed off by the NHS from a clinical and financial standpoint. Individuals can choose to manage their PHBs in different ways depending on the level of financial responsibility they wish to take.

## The evidence supporting the roll out of personal health budgets

There is some evidence for the impact of personal budgets on people with mental health problems in social care, but this is limited. A recent systematic review of 15 studies in this area found mainly positive outcomes.^4^ However, owing to methodological limitations, the findings were judged to be insufficiently robust and not adequate to inform policy and practice.

There is some evidence for the value of PHBs. The national personal health budget evaluation was based on an independent, 3-year longitudinal trial conducted by the Personal Social Services Research Unit (PSSRU), involving a total of just over 2000 people across treatment and control groups and a mixed-methods design with randomisation in some, but not all, local areas.^5^ Outcomes for the PHB and control groups were compared at a target 12th month after initial recruitment. The total sample size was adequate and provided sufficient statistical power. The subgroup analyses for individual health conditions had lower numbers and consequently less power. Participants had a range of health conditions, with 412 experiencing mental health problems. The ‘mental health conditions’ group was not homogeneous and contained a broad range of conditions and severities. The average annual PHB for mental health was £3602.

Overall, the evaluation found that individuals with a personal health budget reported higher levels of care-related quality of life and psychological well-being than those receiving care as usual. PHBs were cost-effective for people with mental health problems and those receiving NHS continuing healthcare, but cost analyses for those with other health conditions were inconclusive owing to small subsample sizes. Those with mental health conditions had lower indirect costs as a result of using fewer in-patient, emergency and GP services. Consequently, personal health budgets were found to provide greater net benefits than conventional services for those with mental health problems. Overall costs for the PHB group showed a 12% decrease at follow-up compared with the 8% increase in costs seen in the control group. Importantly, the way in which PHBs were implemented was found to have an effect on individual outcomes. Offering PHBs so that individuals were able to choose how their budget was spent and managed had a positive impact on outcomes. One restrictive model of implementation used for 18% of the overall PHB sample resulted in less positive impacts for PHB holders than for the control group.^5^

Other studies, including evidence from similar programmes in the USA, also report positive outcomes for individuals, but these are descriptive or pre/post studies.^6^

## Debating personal health budgets

Opinion on PHBs is divided: there are opportunities, but also potential risks. The rest of this article presents five such aspects. In each case, we set out the opportunities and risks and discuss how risks can be managed. Case studies are used to illustrate each point of debate.

### Personal health budgets and the role of evidence-based medicine

PHBs can be spent in ways that do not conform to the current understanding of evidence-based medicine. Individuals are not restricted to treatments that are approved by the National Institute for Health and Care Excellence (NICE). The flexibility of a personal health budget presents a clear opportunity for the NHS to respond to each individual's needs rather than expecting individuals to fit into commissioned services. It is these additional inputs and supports that are often crucial in determining a person's recovery (Box 1). This can be particularly valuable for patients for whom current therapeutic options have not proved successful and who may otherwise disengage from services. Furthermore, literature on the self-management of long-term conditions highlights the importance of individual engagement which PHBs can facilitate.^7^

However, there is also a risk that by not following NICE guidelines, resources are poorly spent and care is either not effective or, at worst, harmful. Important questions remain about how clinicians weigh up the pros and cons of alternative purchases such as a holiday in place of respite and evaluate whether such choices genuinely meet needs. Working with PHBs may necessitate different clinical skills from those required by evidence-based medicine and these new conversations could reshape the doctor/patient relationship.

### Balancing individual choice and risk

Supporting individuals to exercise choice using a PHB can be an effective means of increasing their sense of personal control and opportunities. Furthermore, engaging individuals closely in the development of their care plan can help facilitate their management of risk and safety. Planning PHBs involves working in partnership with individuals to identify risks and how they can be managed safely to achieve the outcomes desired by patients. All PHB plans have to be approved by a clinician and plans should only be signed off if they fully address risk and identify contingencies.

**Box 1** Case study 1: Personal health budget used for additional supports

Alex suffered a stroke which left him with a mild physical and cognitive disability and very anxious and depressed. He had become very fearful of bad news arriving in the post and therefore tended to leave letters unopened and bills unpaid. He used his personal health budget to hire a personal assistant to help him manage his post and other administrative issues and to monitor his medications and diet. He also made several one-off purchases. He bought a satellite navigation device to help him drive without getting lost because the stroke had affected his short-term memory. This enabled him to be an active part of local stroke groups and to drive others to meetings, giving him a renewed sense of purpose. He bought a tablet computer to rebuild his confidence and IT skills. Finally, he bought a drum kit as an alternative to physiotherapy and one that he finds a lot more fun. He attends weekly drum lessons rather than regular physiotherapy. Since getting a PHB he has reduced his use of the community mental health team to three times a year

At the same time, by making different choices from those clinicians would make on their behalf, PHBs can allow individuals to make choices that would increase rather than mitigate their symptoms or put them at greater risk (Box 2). For example, individuals may choose to use complementary therapies that are unproven rather than traditional talking therapies or untrained personal assistants rather than regulated providers. Approving alternative choices can be perceived to be in conflict with the duties of a doctor as set out by the General Medical Council given the lack of established evidence or quality assurance procedures for many alternatives to clinical care.

### The opportunity for greater prevention or the risk of falling back on the NHS

Through the development of person-centred plans, PHBs provide an opportunity for individuals to better manage their ongoing heath and avoid unplanned use of in-patient and crisis care. This is supported by the national evaluation which found that PHB holders made less use of other NHS services, including in-patient care, than those not using PHBs. The difference in service use amounted to, on average, £3050 a year for those with mental health problems.^5^

However, for those individuals whose choices turn out to have limited effectiveness in their mental health management, there is a risk that they may exhaust their PHB without having their needs met. This could leave them either without the care they need or cost the NHS more overall because they fall back on existing services.

In terms of access to needed care, PHBs do not differ from the NHS as a whole. Individuals who are unsuccessful in treatment, be that traditional or through a PHB, are not denied care. To ensure that the choices individuals make are more likely to be effective, clinicians should be closely involved in the development of PHB plans, adding their clinical expertise to the lived experience of individuals (Box 3).

**Box 2** Case study 2: Croydon's personal health budget pilot for substance misuse treatment and recovery

In the evaluation of the Croydon pilot, the lead health professional described how conflict between the choice and wants of service users and the professional opinion of staff played out in one particular case:

‘We did have one client who, when he looked at the cost of what he was recommended for, in-patient detoxification, was surprised at how much it cost. His instinct was immediately to minimise the amount of money that was spent on his medical intervention because he wanted to spend more money on other aspects of his recovery. That led us into a difficult situation and he did relapse and ended up needing another detox, but again only wanted another short detox. So we had that issue about “it's my budget, it's my money”.’

In the end, the PHB for the individual was stopped.^8 p.38^

### Balancing individual choice, equity and efficiency

PHBs are based on a transparent allocation of resources at the individual level that seeks to protect equity within the NHS while allowing individuals greater choice. The approach underpinning PHBs is to maximise outcomes for each individual rather than ensuring that each person receives the same service. Encouragingly, the national evaluation found no differences in the impact of PHBs by gender, ethnicity or income.

The concerns for people who lack capacity are the same for PHBs as for other decisions made for this group – how to make sure that choices are being made in someone's best interests. Family members may effectively act as representatives for individuals who lack capacity or support those with capacity to access a PHB. Those with fluctuating capacity may be encouraged to plan for the future when they are well. Third parties may hold the budget where there are concerns about financial exploitation by family members. As commissioners and clinicians have to approve plans, a plan which is not in the best interests of the patient would not be approved.

There is a risk that PHBs create long-term dependency and a sense of entitlement to support rather than the value of the PHB being reduced as an individual recovers. Furthermore, the expansion of PHBs will have a knock-on effect on the wider service system. If enough individuals use their PHB to make different choices, it may be difficult to maintain a service such as a community mental health team for those who want to continue to use it. This is of particular concern because PHBs are to be implemented from within existing funding and services are already struggling with current levels of resources. There is a risk that those who lose out will be the most vulnerable, whereas those who are better able to choose go elsewhere.

**Box 3** Case study 3: Using a personal health budget to fund psychotherapy

Mary was eligible for a PHB as part of her trust's community mental health pilot. She has depression, anxiety and a personality disorder and has been using mental health services for 10 years. In the year prior to the pilot, she had 18 overnight in-patient stays, three respite stays and 49 contacts with professionals from the community mental health team.

The main thing that Mary wanted to do differently with her PHB was to access long-term psychotherapy as and when she felt she needed it rather than in a 12-week block. A short course of NHS psychotherapy in the past had started to work for her so she negotiated to reduce the input from her care coordinator and psychiatrist and used her PHB to support further engagement in private psychotherapy.

After a year of having a PHB, Mary had only taken one overdose, had reduced her medications, was seeing her care coordinator less often and had started to reconnect with her children and grandchildren.

### Freeing up psychiatrists to focus on clinical care or increasing bureaucracy and workload

The experience of many clinicians working with personal budgets in social care is that they have increased bureaucracy and have made it more difficult to access services for individuals. In part, this is because personal budgets have been implemented at the same time as significant cuts to social care funding and in social care they are still subject to means testing. Critics of PHBs argue that they pose similar risks to the NHS, leading to overly complex processes, bureaucracy and additional costs.^9^

Personalisation does not necessitate greater bureaucracy or work for psychiatrists. Non-clinical staff can support individuals to develop a care plan, seeking input from psychiatrists rather than psychiatrists taking a lead. In fact, experience has shown that non-clinical brokers can be better placed to support individuals to think differently (Box 4).^10^

However, to limit bureaucracy, two things matter. First, there needs to be adequate investment in the infrastructure for PHBs, particularly the necessary support for care planning and avoidance of exploitation. The national evaluation estimated this to be on average £146 000 per clinical commissioning group in the first 2 years, with costs reducing over time.^11^ Second, it is essential to establish clear local guidelines, which can reduce the occurrence of arbitrary decision-making. Decisions should be made with the individual's needs in mind, but the lack of established guidelines can allow prejudicial decisions to creep in.

## Conclusions

Personal health budgets present an opportunity to improve outcomes for those with long-term mental health conditions. When well implemented to offer choice and flexibility, they offer one potentially effective tool for facilitating people's personal recovery, offering the chance of better outcomes for individuals at lower overall cost to the NHS. However, they are not without their risks and challenges, particularly to the long-term sustainability of existing community-based services. Greater clarity from government about the nature and timetable for the roll-out of PHBs in mental health will be essential. Full engagement with psychiatrists will be critical to the success of PHBs but the profession has so far not been significantly involved in shaping the policy or its implementation. We invite you to continue the debate.

**Box 4** Case study 4: Using a social care budget for external support

Paul is a man in his 30s with obsessive-compulsive disorder and Asperger syndrome. He had found it very difficult to be socially active, but with the support of community mental health team psychiatry, psychology and occupational therapy he began to access groups for people with Asperger syndrome. Although he became more engaged in the community, it created challenges for him as he encountered difficult feelings that he interpreted as mental health crises and began using mental health services more frequently. He used a social care budget to hire a support worker from the National Autistic Society who helped him discuss, understand and manage these feelings as they arose. His use of mental health services has subsequently reduced and he also feels happier.
